# Multi-Pixel Simultaneous Classification of PolSAR Image Using Convolutional Neural Networks

**DOI:** 10.3390/s18030769

**Published:** 2018-03-03

**Authors:** Lei Wang, Xin Xu, Hao Dong, Rong Gui, Fangling Pu

**Affiliations:** 1 School of Electronic Information, Wuhan University, Wuhan 430079, China; wanglei2016@whu.edu.cn (L.W.); donghao@whu.edu.cn (H.D.); ronggui2013@whu.edu.cn (R.G.); flpu@whu.edu.cn (F.P.); 2 Collaborative Innovation Center of Geospatial Technology, Wuhan University, Wuhan 430079, China

**Keywords:** Gaofen-3, PolSAR image classification, convolutional neural networks, multi-pixel classification, fixed-feature-size

## Abstract

Convolutional neural networks (CNN) have achieved great success in the optical image processing field. Because of the excellent performance of CNN, more and more methods based on CNN are applied to polarimetric synthetic aperture radar (PolSAR) image classification. Most CNN-based PolSAR image classification methods can only classify one pixel each time. Because all the pixels of a PolSAR image are classified independently, the inherent interrelation of different land covers is ignored. We use a fixed-feature-size CNN (FFS-CNN) to classify all pixels in a patch simultaneously. The proposed method has several advantages. First, FFS-CNN can classify all the pixels in a small patch simultaneously. When classifying a whole PolSAR image, it is faster than common CNNs. Second, FFS-CNN is trained to learn the interrelation of different land covers in a patch, so it can use the interrelation of land covers to improve the classification results. The experiments of FFS-CNN are evaluated on a Chinese Gaofen-3 PolSAR image and other two real PolSAR images. Experiment results show that FFS-CNN is comparable with the state-of-the-art PolSAR image classification methods.

## 1. Introduction

Synthetic aperture radar (SAR) is one of the most important methods of earth observation. It has the advantages of working under all weather conditions, large scope and certain penetration capacity. Modern SAR systems can provide polarimetric SAR (PolSAR) images by emitting and receiving fully polarized radar waves [1]. In recent years, PolSAR has developed rapidly in China. With the launching of the Chinese Gaofen-3 (GF-3) satellite on 10 August 2016, the ability of earth observation of China is improved significantly. GF-3 carries a C-band SAR sensor with different polarizations and operates in 12 different working modes, so it can provide all kinds of polarization images, including single-, dual- and quad-polarization images. GF-3 will greatly help the study of SAR image processing in the next few years.

PolSAR image classification is one of the most important applications in PolSAR image processing, where each pixel in the PolSAR image is assigned to one class. It plays an important role in urban planning, agriculture, disaster prevention and so on [2,3,4]. The methods for PolSAR image classification can be divided into two main categories: one is the traditional statistical modeling [5,6] and the other is the machine learning. For long time the machine learning methods for PolSAR image classification are mainly non-neural machine learning [7] methods, such as support vector machine (SVM) [8] and random forest [9]. These methods have achieved good results [10,11], but the classification accuracy of non-neural machine learning methods depends on the discrimination of feature representation, which always requires designing and tuning manually. The handcrafted features need long time research and rich experience of PolSAR image processing. It is difficult and time consuming to extract discriminative features manually for the non-neural machine learning methods.

Deep learning [12] is a branch of machine learning. Different from the non-neural machine learning methods, deep learning can extract discriminative features automatically. In recent years, deep learning has developed rapidly and the methods based on deep learning surpass the benchmarks again and again in optical image classification [13], image segmentation [14], natural language processing [15], speech recognition [16] and so on.

In PolSAR image processing field, more and more deep learning methods were applied to PolSAR image despeckling, segmentation, target recognition and so on. Wang et al. [17] proposed an image despeckling CNN for automatically removing speckle from the noisy SAR images. Duan et al. [18] used the wavelet to improve the convolutional layer and pooling layer. They proposed a convolutional-wavelet neural network (CWNN) for PolSAR image segmentation. The segmentation result of CWNN was used with a superpixel approach and a Markov random field approach to produce the final segmentation map. In [19], a displacement- and rotation-insensitive deep CNN was trained on moving and stationary target acquisition and recognition (MSTAR) dataset for SAR automatic target recognition and the proposed CNN could achieve high accuracy in three subsets with different displacement and rotation settings.

Deep learning is also widely used in PolSAR image classification. All kinds of deep learning models, such as Deep Belief Networks (DBN) [12], Auto Encoders (AE) [20], Convolutional Auto Encoders (CAE) [21] and CNN [22], have achieved great results. Zhao et al. [23] combined the ensemble learning with a DBN in an unsupervised manner to learn discriminative features. Xie et al. [24] proposed a new method named WAE and WCAE, which combined the Wishart distance measurement into the training process of the AE and CAE. Compared with AE and CAE models, WAE and WCAE models could achieve higher classification accuracy. In [25], the covariance matrix of a PolSAR image was converted into a normalized 6-D real feature vector. Then the six-channel real feature vector was fed into a CNN for PolSAR classification. Gao et al. [26] proposed a dual-branch deep CNN to realize the classification of PolSAR images. So far, CNN is the state-of-the-art approach for image classification. In the ImageNet ILSVRC image classification contest, the CNN-based models won the first prize from 2012 to 2017.

In PolSAR image classification, the deep learning methods are only used to classify one pixel each time [23,24,25,26,27]. In general, if we classify a pixel, the low level features generated from the neighborhood of the pixel are input into a deep learning model, and then the classification result of the pixel is output from the model. If an entire PolSAR image needs to be classified, the neighborhood of each pixel should be input to the deep learning model one by one. First, it is slow to classify all the pixels of a PolSAR image one by one. Second, all the pixels are classified independently, so the interrelation of land covers is not used. Although the neighborhood of a pixel is used for classification, the land covers in the neighborhood are unknown. CNNs only use the data from the neighborhood of a pixel, but the inherent relationship of different lander covers is ignored. For example, there are two pixels that are next to each other. The CNNs classify the first pixel to water. Then the CNNs classify the second pixel independently, no matter what the land cover of the first pixel is. Some deep learning methods can generate pixel-wise bidimensional maps of classes [14,28,29], but few of these kinds of methods are applied to PolSAR image classification. We aim to provide a fast and simple CNN for classifying multiple pixels simultaneously in patch level, so a fixed-feature-size convolutional neural network (FFS-CNN) is proposed. FFS-CNN can classify multiple pixels in a patch simultaneously. It is faster than common CNNs when classifying a whole PolSAR image. The predictions of land covers of the pixels in a patch are known to each other. The FFS-CNN is trained to make use of the interrelation of the land covers of the pixels to improve the classification performance.

The rest of this paper is organized as follows. In Section 2, we briefly introduce the framework of CNN and particularly illustrate the structure, implementation and characteristics of FFS-CNN. In Section 3, the classification results of three real PolSAR data, one of which was acquired by GF-3 sensor, are evaluated to verify the performance of FFS-CNN. In Section 4, the factors that affect the performance of FFS-CNN are discussed. Finally, the conclusion is drawn in Section 5.

## 2. Materials and Methods

### 2.1. Framework of the CNN

Typically, the CNN is stacked by convolutional layers, pooling layers, and fully connected layers. All the layers are connected in series and the input data of a layer is the output data of the previous layer. The input data of the first layer is an image or low level features. Because of the deep connected structure, the CNN can extract high level features from low level features. The convolutional layer convolves the feature maps of the previous layer with learnable kernels and puts the results through activated function to generate the output feature maps [30], as shown in the equation below.

(1) $$x_{j}^{l}=f\left(\sum_{i\in M_{j}}x_{j}^{\left(l-1\right)}*k_{ij}^{l}+b_{j}^{l}\right)$$

where $M_{j}$ denotes the input feature map, $k_{i}^{j}$ denotes the convolutional kernel, $b_{j}$ denotes the bias and $x_{j}^{l}$ denotes the output feature map. f(·) is the nonlinear activation function, such as sigmoid function and Rectified-Linear Units (ReLU) [31].

The convolutional layer is usually followed by a pooling layer. The pooling layer can reduce the dimension of the feature map and prevent overfitting. The pooling layer computes a value from a local window of the input feature map. Different pooling layers have different algorithms. The most common used is max pooling, it chooses the max value of a local window as the output. The other pooling layers are avenge pooling, stochastic pooling, Spatial Pyramid Pooling (SPP) [32] and so on.

The fully connected layer is usually on the top of a CNN. It multiplies the input feature maps with learnable weights to generate the output feature maps, which is shown as below

(2) $$x_{j}^{l}=f\left(\sum_{i\in M_{j}}x_{j}^{\left(l-1\right)}\cdot w_{ij}^{l}+b_{j}^{l}\right)$$

where $M_{j}$ is the input feature, $x_{j}^{l}$ is the output feature, $w_{ij}^{l}$ is the learnable weights and $b_{j}^{l}$ is the bias. f(·) is the nonlinear activation function, too. In the task of classification, the fully connected layer can extract a 1D feature vector and puts the vector to the softmax layer. The dimension of the feature vector is the number of the classes.

In the task of classification, softmax layer is the classifier of a CNN. It is defined as follows

(3) $$\sigma_{i}\left(z\right)=\frac{exp(z_{i})}{\sum_{j=1}^{m}exp\left(z_{j}\right)},i=1,2,\cdots ,m$$

where $z_{i}$ is one of the prediction of previous layer. $\sigma_{i}\left(z\right)$ is a nonnegative and normalized value, which is the probability of class *i*. The softmax layer calculates the probabilities of all classes and the class with max probability is the final classification result.

**Figure 1:**
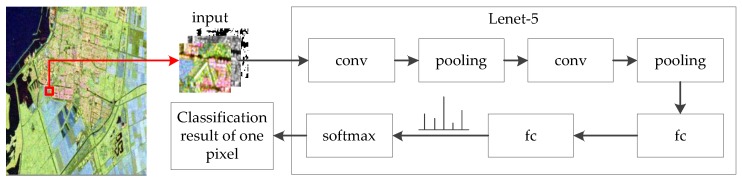
PolSAR image classification architecture based on Lenet-5.

The most common used CNN for PolSAR image classification is Lenet-5 [25] or improved Lenet-5 [26,27]. Other very deep CNNs such as AlexNet [13], VGGNet [33], GoogLeNet [34] and ResNet [35] are suitable for images of large size. The CNNs need many samples to train the weights but the PolSAR images are always not sufficient from one SAR sensor. In PolSAR image classification field, the PolSAR images are split into a significant amount of patches and some patches are randomly selected as the training samples of CNNs. The size of the patches is always small, such as 7×7, 9×9, 15×15. When the small patches pass through multiple convolutional layers and pooling layers, the size of final feature map is even smaller and may become zero. Therefore, more time is needed to tune the parameters of these very deep CNNs mentioned above to satisfy the requirement of the PolSAR image classification task. Lenet-5 is composed of 2 convolutional layers, 2 pooling layers, 2 fully connected layers and a softmax layer, as shown in Figure 1. Figure 1 also illustrates the architecture of Lenet-5 for PolSAR image classification. The input is the neighborhood of a pixel and the output is the classification result of the pixel.

In optical image processing, some CNN models can output pixel-wise predictions simultaneously for all the pixels of an image. Take [14] as example, the authors proposed fully convolutional neural networks (FCN) for semantic segmentation. With a well adapted classifier for dense prediction, FCN can output pixel-wise bidimensional map of an input image.

Generally, most CNN models for classification, including Lenet-5 and FCN, can be divided into two parts, which are the feature extraction part and the classifier. According to different deep learning algorithms, the architectures of feature extraction parts are different, such as Lenet-5, AlexNet and ResNet. The most common used classifier for CNN is softmax. The softmax not only can output one class, such as Lenet-5, but also can output a bidimensional map of classes, such as FCN. FFS-CNN can also be divided to the feature extraction part and classifier. The feature extraction part of FFS-CNN makes some reference to Lenet-5 and classifier of FFS-CNN is the same as FCN. The detailed structure of FFS-CNN is illustrated in Section 2.2.

### 2.2. Fixed-Feature-Size Convolution Neural Networks

**Figure 2:**
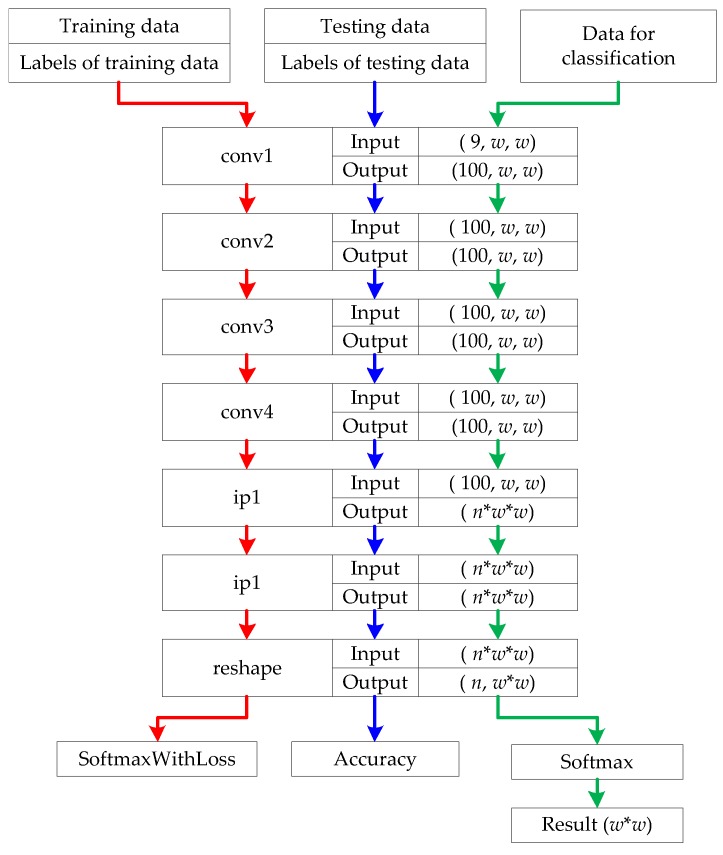
The structure of FFS-CNN. The red arrows and blue arrows show the training procedure and green arrows show the classification procedure.

The structure of FFS-CNN is shown in Figure 2. As the size of input patches is small, the feature extraction part of FFS-CNN should as simple as Lenet-5. The feature extraction part of FFS-CNN contains 4 convolutional layers, 2 fully connected layers, and a reshape layer. The classifier of FFS-CNN is softmax, which can produce pixel-wise predictions for all pixels in a patch. The kernel size of each convolutional layer is 3×3, stride is 1 and pad is 1. So for each convolutional layer, the size of input feature maps and output feature maps is the same. The size of input patches is denoted by w×w and the number of channels is 9. Firstly the input patches pass through 4 convolutional layers, the size of output feature maps of the fourth convolutional layer is w×w with 100 channels. Then the feature maps pass through 2 fully connected layers and the output feature is a 1D vector. The size of the 1D vector is (w×w×n), where *n* denotes the number of classes and the expression in parentheses represents a number. In order to match the input format of the softmax layer to classify all pixels simultaneously, the 1D feature vector needs to be reshaped to 2D feature matrices. The size of output feature matrices of the reshape layer is n×(w×w). Finally the softmax layer uses the feature matrices to calculate the probability of each class for the pixels of w×w and the class with max probability is the classification result of each pixel.

There are three kinds of input data, which are training samples, testing samples and the data for classification. In Figure 2, the red arrows and blue arrows show the training procedure and green arrows show the classification procedure. The training samples and their labels are used to train the weights of all layers through back propagation (BP) and gradient descent. The testing samples and their labels are checked during training to monitor the progress and coarse accuracy of the model, but are never used for gradient descent. In the procedure of classification, all patches from a PolSAR image are input to the trained model to get the classification results of the pixels in the image. In the training procedure, the image patch and the labels of all pixels in the patch are used to train the FFS-CNN. In this way, the FFS-CNN can learn the interrelation of land covers in the patch. For example, if the neighboring pixels of a pixel are all water, then this pixel most probably is water.

As we mentioned in Section 2.1, the feature extraction part of FFS-CNN makes some references to Lenet-5. In order to keep the feature size invariable, the two pooling layers of Lenet-5 are changed to convolutional layers. The parameters of convolutional layers and fully connected layers are also specially designed. In [14], the softmax layer is used to classify multiple pixels, so FFS-CNN also use the softmax layer to classify the pixels in a patch simultaneously.

**Figure 3:**
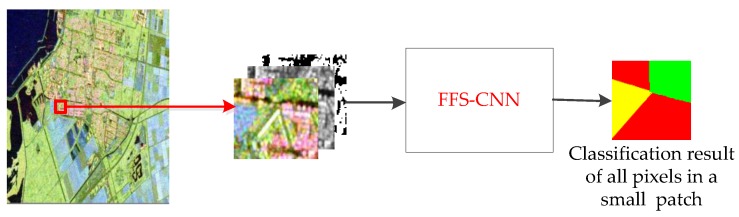
Multi-pixel simultaneous classification of PolSAR images.

The FFS-CNN has two main characteristics:

1. The FFS-CNN implements the multiple pixels simultaneous classification in a patch, which is illustrated in Figure 3. The number of land covers in a patch is equal to or smaller than the number of classes *n*. It is not hard to classify w×w pixels to no more than *n* classes through CNN. Because FFS-CNN can classify the multiple pixels simultaneously and its structure is simple, it is much faster than Lenet-5 when classifying a whole PolSAR image.
2. FFS-CNN can use the interrelation of different land covers. In the training procedure, FFS-CNN uses the labels of pixels in a patch to learn the interrelation of land covers. In the classification procedure, the interrelation of land covers is used to predict the classes of pixels in a patch.

**Figure 4:**
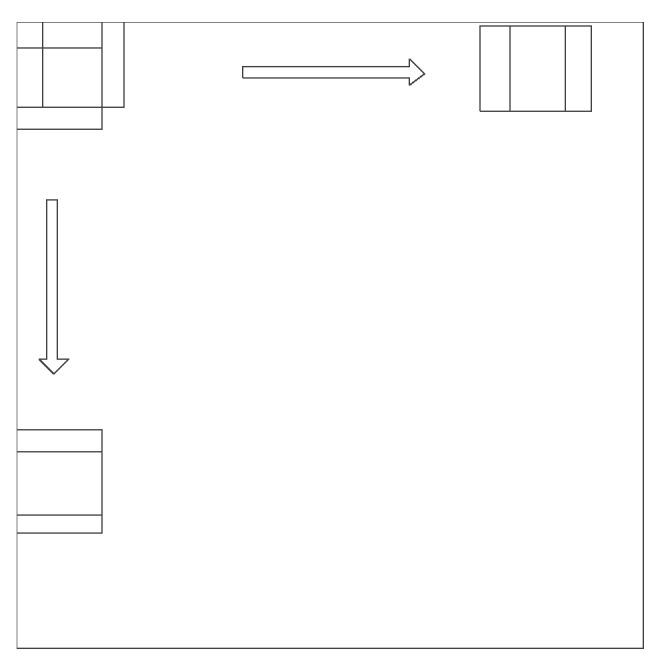
Strategy of 3/4 overlap classification.

Based on the architecture of the FFS-CNN, the pixels in a patch can be classified simultaneously, so the sliding window method is used to classify the entire PolSAR image. In this paper, the window slides w/4 pixels. In other word, about 3/4 pixels are overlapped and will be classified again. Figure 4 shows the strategy of 3/4 overlap classification. Each pixel of a PolSAR image is classified multiple times so the probabilities are averaged. For each pixel, the class of maximum probability is taken as the final classification result. In the video activity recognition section of [36], the authors also averaged the label probabilities across all frames of an entire video to choose the most probable label.

### 2.3. Input Data of FFS-CNN

PolSAR image can be expressed with polarization coherent matrix $T_{3}$. It has the following form.

(4) $$T_{3}=\left[\begin{array}{ccc} T_{11} & T_{12} & T_{13}\\ T_{21} & T_{22} & T_{23}\\ T_{31} & T_{32} & T_{33} \end{array}\right]$$

where $T_{11}$, $T_{22}$, $T_{33}$ are real numbers, the others are complex numbers. $T_{12}$ is the conjugate complex number of $T_{21}$, $T_{13}$ is the conjugate complex number of $T_{31}$ and $T_{23}$ is the conjugate complex number of $T_{32}$. To make full use of the polarimetric information, the matrix $T_{3}$ is used to generate the input data of FFS-CNN. For each pixel, the polarimetric data can be defined as a vector $t_{p}$.

(5) $$t_{p}=\left[T_{11},T_{22},T_{33},Re\left(T_{12}\right),Im\left(T_{12}\right),Re\left(T_{13}\right),Im\left(T_{13}\right),Re\left(T_{23}\right),Im\left(T_{23}\right)\right]$$

**Figure 5:**
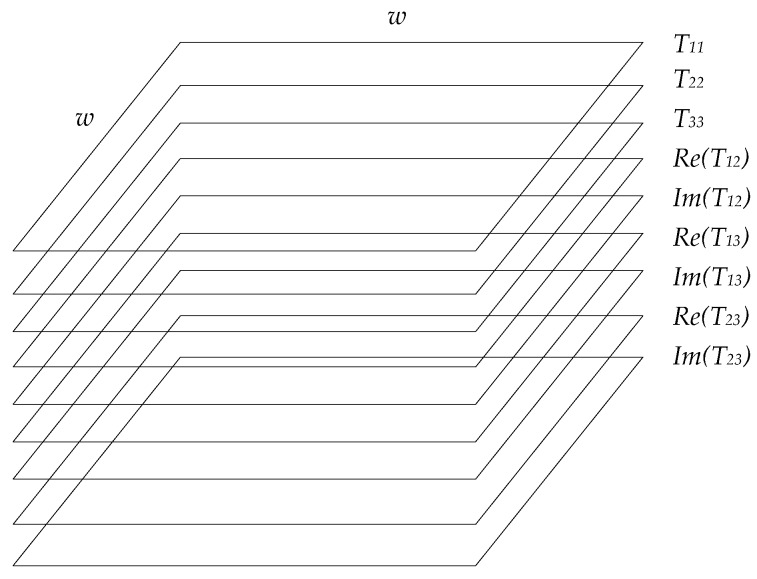
Input matrix of FFS-CNN.

Then the polarimetric data of all the pixels in a patch can be used to generate a matrix as the input of FFS-CNN, which is shown in Figure 5. The dimension of the matrix is 9×w×w . For each channel, normalization is needed.

### 2.4. Materials

Three real PolSAR datasets, including two spaceborne PolSAR images and one airborne PolSAR image, are used to verify the performance of FFS-CNN. The detailed data information is presented in Table 1.

#### 2.4.1. RADARSAT-2 Flevoland Dataset

**Figure 6:**
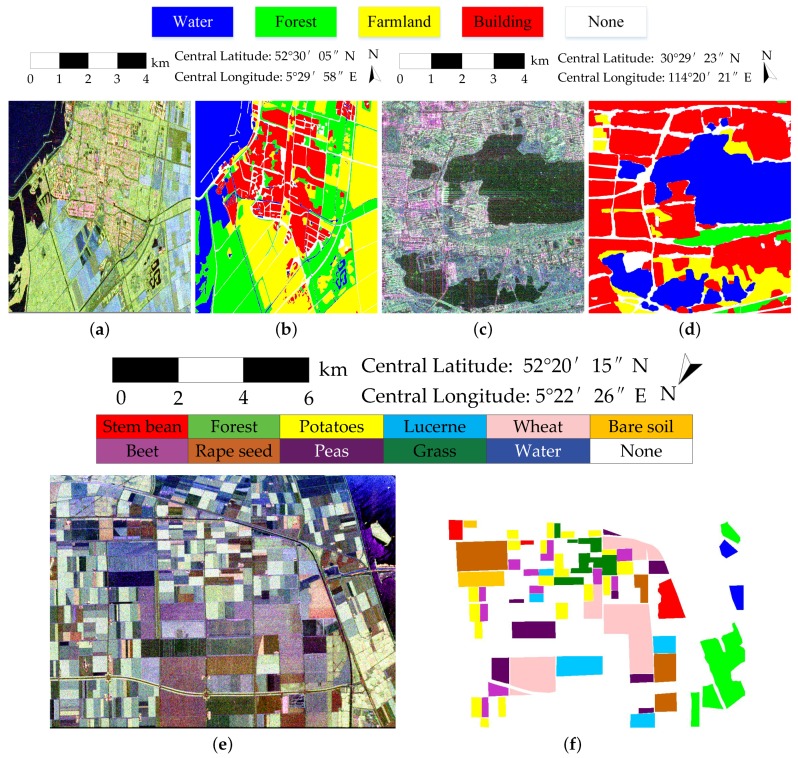
PolSAR images and the ground truth maps for land cover classification. (**a**) Pauli RGB image of RS-2 Flevoland; (**b**) Ground truth map of RS-2 Flevoland; (**c**) Pauli RGB image of GF-3 Wuhan; (**d**) Ground truth map of GF-3 Wuhan; (**e**) Pauli RGB image of AIRSAR Flevoland; (**f**) Ground truth map of AIRSAR Flevoland.

The spaceborne dataset is acquired by the C-band RADARSAT-2 (RS-2) PolSAR system at fine quad-pol mode. It is over Flevoland in the Netherlands, with an image size of 1400×1200 pixels. The spatial resolution is 12 m in range direction and 8 m in azimuth direction. A total of four classes of Flevoland dataset are identified, consisting of water, forest, farmland, and buildings. Figure 6a shows the Pauli RGB image. Figure 6b shows the ground truth map, which was manually created based on very high resolution optical images.

#### 2.4.2. AIRSAR Flevoland Dataset

The airborne dataset is the NASA/JPL AIRSAR L-band four-look fully polarimetric data. The Pauli color-coded image is shown in Figure 6e. This scene also covers over Flevoland, the Netherlands, with an image size of 750×1024 pixels and a spatial resolution of 6×12 m. Since [37], this dataset is widely used in land cover classification with the well-established ground truth map, which is shown in Figure 6f. A total of 11 classes are identified, consisting of eight crop classes, and three other classes of bare soil, water, and forest.

#### 2.4.3. Gaofen-3 Wuhan Dataset

**Figure 7:**
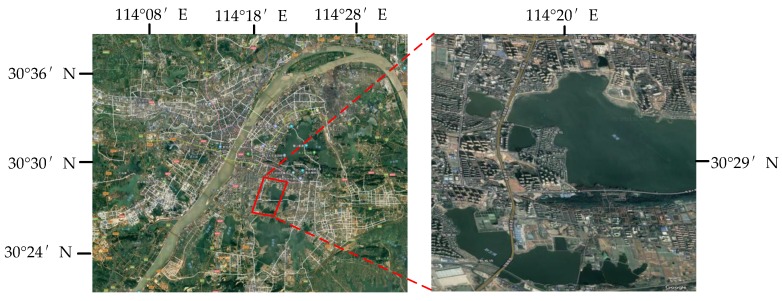
Optical image of Wuhan.

The other spaceborne dataset is acquired by the C-band GF-3 PolSAR system at quad-polarized strip I (QPSI) mode. The scene used in this paper covers over local area of Wuhan, China, with an image size of 1050×1000 pixels and a spatial resolution of 5.20 in range direction and 2.25 m in azimuth direction. It has four classes, which are water, forest, farmland and buildings. The Pauli RGB image and ground truth map are shown in Figure 6c,d. The ground truth map is labeled manually according to the high resolution optical image, which is shown in Figure 7.

For an entire PolSAR image, the sliding window of w×w is used to generate a significant amount of image patches, which can serve as the training and testing samples. For each dataset, the training samples and testing samples are selected randomly from those generated patches but the numbers of training samples and testing samples are a little different. The numbers of samples of the three datasets are introduced in Table 1.

## 3. Results

All the experiments in this paper are based on our deep learning acceleration computing service. The CPU is i7-7700, the graphics card is NVIDIA GTX 1080ti and the RAM is 16G. The system of the computing service is Ubuntu 16.10 and all the CNN models are trained with Caffe [38]. We set the size of input patches *w* to 15. The OA and kappa are used to judge the performances of the models, where OA stands for the overall accuracy and kappa stands for the kappa coefficient. The final classification results of the datasets are used to calculate the OA and kappa.

### 3.1. Results of RS-2 Flevoland

For RS-2 Flevoland, there are two kinds of training and testing simples, which are named Samples 1 and Samples 2. The Samples 1 are randomly selected from the total patches. The whole PolSAR image is used to evaluate the classification results. The Samples 2 are randomly selected from the patches that generated from the top half of the PolSAR image. Only the bottom half of the PolSAR image is used to evaluate the classification result. Because there are no training samples from the bottom half of the PolSAR image, the classification results of the bottom half of the PolSAR image are totally independent from the training samples and can more clearly show the generalization ability of the models. The number of samples in Samples 1 and Samples 2 is the same. In [39], the authors used RS-2 Flevoland dataset to judge the performance of their method. The ground truth map in [39] is different from ours, so the result in [39] can be used as a reference but should not be used as the benchmark to judge the performances of the models.

**Figure 8:**
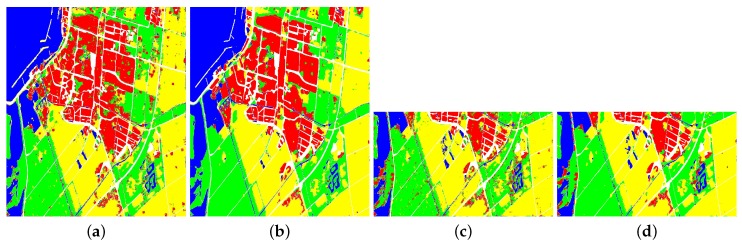
Classification result of RS-2 Flevoland. (**a**) Lenet-5, training with Samples 1; (**b**) FFS-CNN, training with Samples 1; (**c**) Lenet-5, training with Samples 2; (**d**)FFS-CNN, training with Samples 2.

Figure 8 and Table 2 show the classification results and accuracies of RS-2 Flevoland dataset. No matter what kind of the training samples is, the accuracies of 4 classes of FFS-CNN are all higher than Lenet-5. When the training and testing samples are Samples 1, the OA of FFS-CNN is 3.44% higher than Lenet-5. When the training and testing samples are Samples 2, both the OAs of FFS-CNN and Lenet-5 become lower but the OA of FFS-CNN is still 3.92% higher than the OA of Lenet-5. Hence, FFS-CNN can learn more discriminative feature representation than Lenet-5. The OAs of FFS-CNN also higher than the method in [39].

### 3.2. Results of AIRSAR Flevoland

Because the labeled land covers of AIRSAR Flevoland are irregular, they cannot be divided into 2 parts simply for selecting samples. All the training samples and testing samples are randomly selected from the total generated patches.

**Figure 9:**
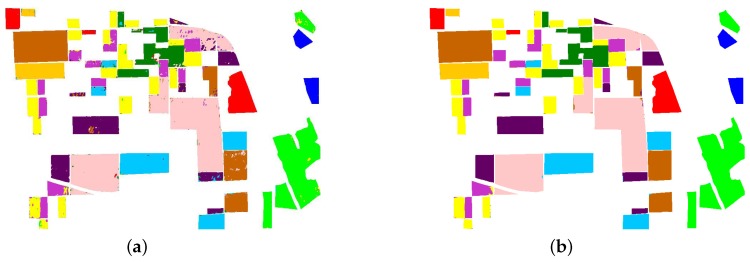
Classification result of AIRSAR Flevoland. (**a**) Lenet-5. (**b**) FFS-CNN.

Figure 9 and Table 3 show the classification results and accuracies of AIRSAR Flevoland. The accuracies of 11 classes of FFS-CNN are all higher than Lenet-5. The OA of FFS-CNN of AIRSAR Flevoland is 2.62% higher than Lenet-5.

The AIRSAR Flevoland dataset is widely used in some other papers, such as [26,27]. In [26], the authors proposed a dual-branch CNN. The dual-branch CNN was compare with PauliRGB-CNN. The PauliRGB-CNN only used the Pauli RGB image as the input. In [27], the authors proposed a complex-valued CNN (CV-CNN). CV-CNN was compared with the real-valued CNN (RV-CNN). The ground truth map of AIRSAR Flevoland in both [26,27] are different from ours. The results can be used as a reference but should not be used as the benchmark to judge the performances of the models. The results are shown in Table 4. The OA of FFS-CNN is the highest.

### 3.3. Results of GF-3 Wuhan

For GF-3 Wuhan dataset, there are also two kinds of samples named Samples 1 and Samples 2, like RS-2 Flevoland dataset. Samples 1 are selected from the total patches. The whole PolSAR image is used to evaluate the classification result. Samples 2 are selected from the patches that generated from the right 1/5 of the PolSAR image. The left 4/5 of the PolSAR image is used to evaluate the classification result. The number of samples in Samples 1 and Samples 2 is the same.

**Figure 10:**
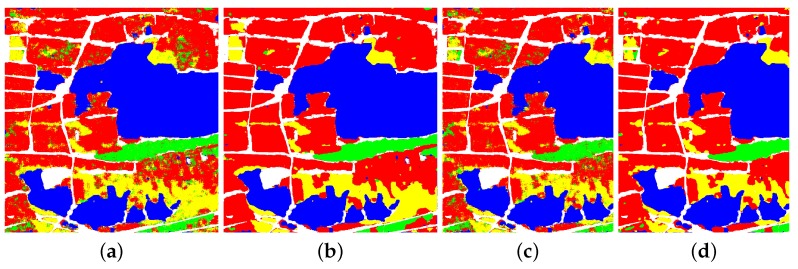
The classification result of GF-3 Wuhan. (**a**) Lenet-5, training with Samples 1; (**b**) FFS-CNN, training with Samples 1; (**c**) Lenet-5, training with Samples 2; (**d**) FFS-CNN, training with Samples 2.

The classification results and accuracies of GF-3 Wuhan are shown in Figure 10 and Table 5. When the training and testing samples are Samples 1, the accuracies of 4 classes of FFS-CNN are all much higher than Lenet-5. The OA of FFS-CNN is 7.00% higher than the Lenet-5. When the training and testing samples are Samples 2, the accuracies of 4 classes of FFS-CNN are also higher than Lenet-5 and the OA of FFS-CNN is 4.85% higher than Lenet-5. It again illustrates that FFS-CNN can learn more discriminative feature representation than Lenet-5.

## 4. Discussion

The feature extraction part of FFS-CNN makes some references to Lenet-5 but the performance of FFS-CNN is much higher than Lenet-5. Moreover, the Samples 2 are totally independent from the evaluation of PolSAR images and the results of FFS-CNN are also better than Lenet-5. This illustrates that FFS-CNN can learn more discriminative feature representation. Three factors play important roles in the good results of FFS-CNN. First, the feature size of all layers in FFS-CNN is fixed. Second, FFS-CNN is trained to use the interrelation of land covers in a patch. Third, the sliding window classification strategy is used to classify a whole PolSAR image. In the following subsections, we will discuss how the three factors affect the classification accuracy of FFS-CNN.

### 4.1. Discussion on Feature Size

**Figure 11:**
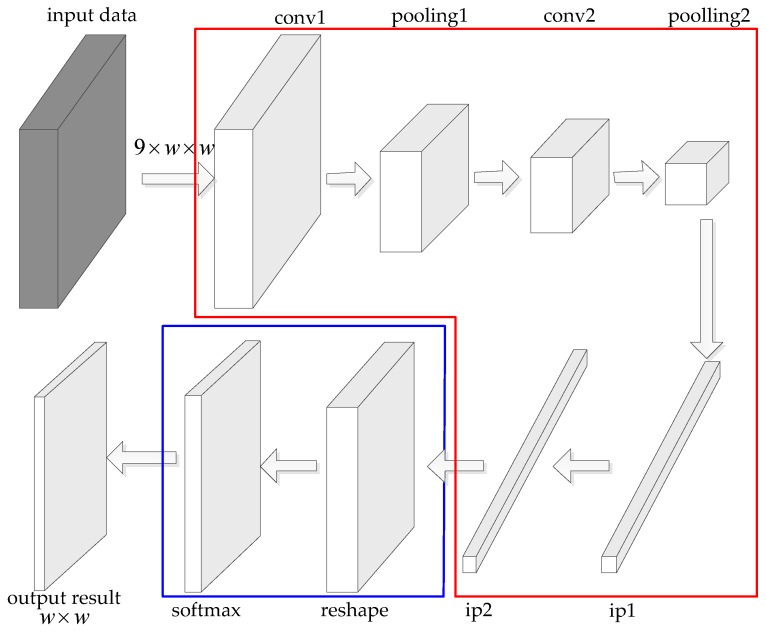
Decreased-feature-size CNN.

The feature size of each layer of FFS-CNN is invariant. In order to compare the performance with the CNN of which the feature size decreases, the second and fourth convolutional layers of FFS-CNN are changed to pooling layers. We call it decreased-feature-size CNN. The structure is shown in Figure 11. The layers in red box are the same as Lenet-5 while the layers in blue boxes are the same as FFS-CNN, so the decreased-feature-size CNN can also classify multiple pixels simultaneously in a patch. If the size of input patches is 15×15, then the size of the output features of the second pooling layer is 4×4. Because all the pixels are classified by softmax layer, the size of input features of the softmax layer should be n×15×15. The first fully connected layer upsamples the features to the size of (n×15×15). Other experiment parameters are the same as the experiments in Section 3.

The classification results are shown as Table 6. For all datasets, the OAs of FFS-CNN are much better than the decreased-feature-size CNN. There are two reasons. First the feature size is fixed, FFS-CNN has more weights in convolutional layers. Second, FFS-CNN do not need to upsample the features. FFS-CNN can learn more discriminative feature representation and get better classification results than decreased-feature-size CNN.

### 4.2. Discussion on Interrelation of Land Covers

FFS-CNN can use the interrelation of the land covers in a patch. In this additional experiment, special patches are randomly selected from the total generated patches as the training samples. The land covers of the pixels in each special patch are the same. We call it one-class training samples. In this way, we can remove the effect of the interrelation of different land covers. In Section 3, the land covers of the pixels in each training patch are different, so they are called multi-class training samples. Other experiment parameters are the same as the experiments in Section 3.

Table 7 shows the experiment results of FFS-CNN and Lenet-5 of different training data types. For the RS-2 Flevoland dataset, when the Lenet-5 and FFS-CNN are trained with one-class training samples, the OA of Lenet-5 is decreases by around 1% and the OA of FFS-CNN decreases by around 3%. The OA of FFS-CNN decreases much more than Lenet-5. The Lenet-5 classifies one pixel each time and does not use the interrelation of land covers, so the OA only decreases a little. The interrelation of different land covers is removed from the one-class training samples, so FFS-CNN cannot learn the interrelation of land covers and cannot use the interrelation of land covers to classify the pixels in a patch. Hence, the OA of FFS-CNN decreases a lot. For the AIRSAR Flevoland dataset, the OAs of Lenet-5 and FFS-CNN are both decrease by around 1–2% when the models are trained with one-class training samples. The OA of FFS-CNN still decreases more than Lenet-5. For the GF-3 Wuhan dataset, the OA of Lenet-5 increases but the OA of FFS-CNN decreases by 1.38% when the models are trained with one-class training samples. This is again illustrates that the interrelation of different land covers can improve the accuracy of FFS-CNN but makes no sense to Lenet-5. From the classification results of the three datasets we can see that FFS-CNN can use the interrelation of land covers to the improve the multiple pixels classification results.

### 4.3. Discussion on Overlap Ratio

In Section 3, Section 4.1 and Section 4.2, the overlap ratio is 3/4. In order to discuss the effect of overlap ratio, in this section the overlap ratios are set to 0, 1/4, 1/2, 3/4, 7/8, where 0 stands for on overlap. The classification time is also recorded based on the hardware and software platform of our deep learning acceleration computing service with no special optimization. Only one patch is input to the models each time.

**Figure 12:**
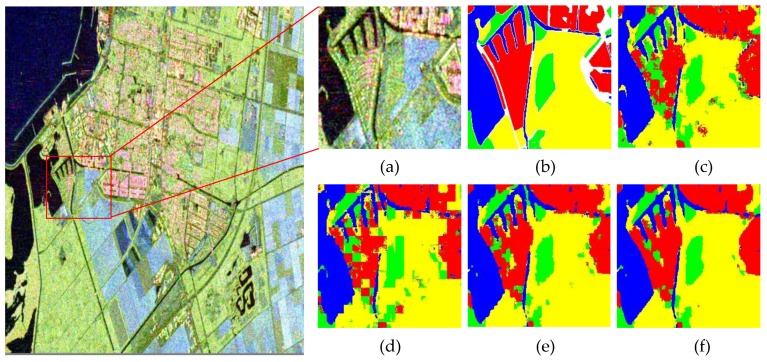
Classification result of local area of RS-2 Flevoland. (**a**) Pauli RGB image; (**b**) Ground truth map; (**c**) Lenet-5; (**d**) FFS-CNN, no overlap; (**e**) FFS-CNN, 1/2 overlap; (**f**) FFS-CNN, 3/4 overlap.

Table 8 illustrates the OAs of the three datasets. Especially, Figure 12 shows the classification results of the local area of RS-2 Flevoland, which can more clearly shows the details of classification results. In the situation of no overlap classification strategy, the OA of RS-2 Flevoland of FFS-CNN are almost the same as the Lenet-5, but the classification result of FFS-CNN has mosaic effect, which can be seen in Figure 12. For the AIRSAR Flevoland dataset, the OA of FFS-CNN is a little lower than Lenet-5. There are two reasons. First, Lenet-5 uses one input data to only predict the class of one pixel while FFS-CNN uses the same input data to predict the classes of w×w pixels. Second, the performance of FFS-CNN depends on the interrelation of land covers in a patch, which is discussed in Section 4.2. The labeled pixels of AIRSAR Flevoland are scattered and FFS-CNN cannot learn enough interrelation of land covers, so FFS-CNN loses the edge to Lenet-5 and the OA of FFS-CNN of AIRSAR Flevoland is a little lower. For the GF-3 Wuhan dataset, the OA of FFS-CNN is much better than Lenet-5. In the situation of 1/2 overlap classification strategy, the classification results of FFS-CNN are improved significantly and all much better than Lenet-5. In the meanwhile, the mosaic effect is almost disappeared. In the situation of 3/4 overlap classification strategy, the OAs of FFS-CNN increase further and the classification results are very fine.

Table 9 illustrates the classification time of the three datasets. First, when the overlap ratio is 1/2, the OAs of FFS-CNN are much better than Lenet-5 while the classification times of FFS-CNN are almost ten times less than Lenet-5. FFS-CNN is much faster than Lenet-5. Second, when the overlap ratio is increases from 0 to 3/4, the OAs increase dramatically and the classification time increases a lot, too. The classification times of FFS-CNN are still about a half of the classification times of Lenet-5. FFS-CNN is still faster than Lenet-5. When the overlap ratio increases to 7/8, the OAs only increase slightly, but the classification times increase almost 10 times more. Therefore, if we give priority to the classification speed, overlap ratio can be set to 1/2. If we prefer to the classification accuracy, then the overlap ratio of 3/4 is the best choice. As long as the overlap ratio is less than 3/4, FFS-CNN is much faster than Lenet-5 for classification a whole PolSAR image. There is no need to increase the overlap ratio over 3/4.

### 4.4. Visualization of Outputs of Convolutional Layers

In this section we will visualize the output feature maps of the convolutional layers. The output features of convolutional layers have multiple channels and no standard method is proposed to visualize the features in PolSAR image classification. In this paper, all channels of the features are simply added up and the values are mapped to the range from 0 to 255, so the features are shown as gray images.

**Figure 13:**
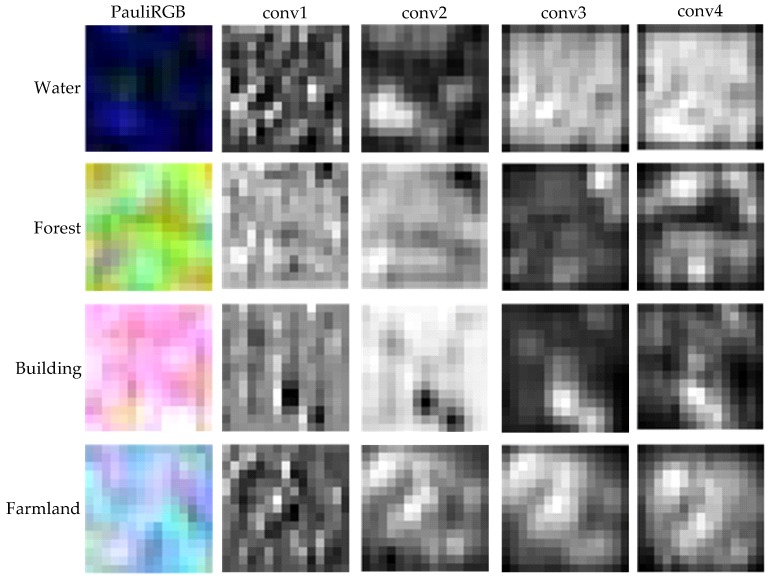
The Pauli RGB images and visualizations of four convolutional layers of water, forest, building and farmland.

We select four one-class training patches from RS-2 Flevoland dataset as the input of FFS-CNN. The size of the patches is 15×15. Figure 13 shows the Pauli RGB images and output feature maps of four convolutional layers. The blurry textures of output feature maps of the fourth convolutional layers are consist with the Pauli RGB images and the output feature maps of different land covers have distinct differences.

**Figure 14:**
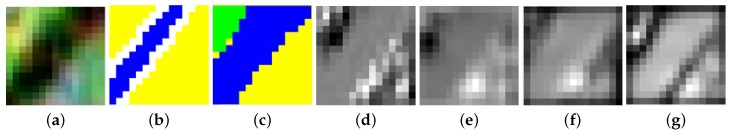
The visualization of multiple land cover data. (**a**) Pauli RGB image; (**b**) Classification result; (**c**) Ground truth map; (**d**)Output of the conv1; (**e**) Output of the conv2; (**f**) Output of the conv3; (**g**) Output of the conv4.

We also select a multi-class training patch from RS-2 Flevoland dataset as the input of FFS-CNN. The size of the patch is also 15×15. Figure 14 shows the Pauli RGB image, ground truth map, classification result and the output feature maps of the four convolutional layers. The ground truth map has 2 land covers while the actual classification result has 3 land covers. There are three areas in the Pauli RGB images. The water area is in the middle and two farmland areas are on either side of the water area. The output feature maps of four convolutional layers are all have distinct three areas. The three areas are finally classified to forest, water and farmland. Compare with the ground truth map, the classification result of top-left corner is not correct, but it is consistent with the Pauli RGB image.

From the above analysis, the FFS-CNN can extract discriminative features of different land covers for the classification of multiple pixels in a patch simultaneously.

### 4.5. Future Works

Although FFS-CNN achieves good classification results for the three dataset, FFS-CNN also has a disadvantage. When classifying a whole PolSAR image with no overlap classification strategy, the accuracies are only comparable with Lenet-5 and the results have mosaic effect. For RS-2 Flevoland, the OAs of FFS-CNN and Lenet-5 are almost the same. For AIRSAR Flevoland, the OA of FFS-CNN is a little lower than Lenet-5. For GF-3 Wuhan, the OA of FFS-CNN is higher than Lenet-5. The reason is that the classification results are not continuous between the edges of different image patches.

There are two main research directions to further improve the classification results in the future. First, the conditional random fields (CRF) can be added to FFS-CNN. It is proven that CFR can improve the pixel-wise prediction accuracies of all pixels in an optical image [28], so we believe that CRF can improve the classification results of FFS-CNN as well. Second, more advanced CNN architectures can be introduced to the feature extraction part of FFS-CNN, such as the inception unit and residual unit in GoogLeNet and ResNet, respectively.

## 5. Conclusions

In this paper, the proposed FFS-CNN method can classify all pixels in a patch simultaneously and has achieved great results. The OAs of FFS-CNN of the three real PolSAR image datasets all surpass the OAs of Lenet-5. From the experiments we can get the following conclusions. First, the interrelation of different land covers in a patch is indeed helpful for multiple pixels classification. Second, the relationship between the overlap ratio and classification accuracies is analyzed. When the overlap ratio is 1/2, the classification times are about 10 times less than Lenet-5. When the overlap is 3/4, the classification times are about a half of the classification times of Lenet-5. Especially, when the overlap ratio is 3/4, the classification results of FFS-CNN are the best and are much better than Lenet-5.

## Figures and Tables

**Table 1:** Three real PolSAR datasets used for evaluation.

Parameter	RS-2 Flevoland	AIRSAR Flevoland	GF-3 Wuhan
Sensor	C-band RADARSAT-2	L-band NASA/JPL AIRSAR	C-band GF-3
Imaging Area	Flevoland	Flevoland	Wuhan
Imaging Mode	Quad-pol	∖	QPSI
Imaging time	2008	16-8-1989	1-4-2017
Spatial resolution [Range Azimuth] (m)	12×8	6×12	5.20×2.25
Image size [Range Azimuth] (pixel)	1400×1200	750×1024	1050×1000
Number of total patches	1,680,000	768,000	1,050,000
Number of training patches	50,400	38,390	22,904
Number of testing patches	16,800	7778	7632

**Table 2:** The classification accuracies of RS-2 Flevoland (%).

Method	Water	Forest	Building	Farmland	OA	Kappa
Lenet-5 (Samples 1)	96.00	89.54	89.93	90.18	90.96	0.88
FFS-CNN (Samples 1)	**97.90**	**91.91**	**93.99**	**94.98**	**94.40**	**0.92**
Lenet-5 half (Samples 2)	93.42	80.44	84.15	92.72	87.34	0.81
FFS-CNN half (Samples 2)	94.01	87.22	90.04	94.47	91.26	0.86
Method in [39]	98.65	89.07	73.40	89.42	90.01	N/A

**Table 3:** The classification accuracies of AIRSAR Flevoland (%).

Land Cover	Lenet-5	FFS-CNN
Water	99.73	**100.00**
Forest	97.96	**99.84**
Stem bean	98.80	**99.75**
Potatoes	97.68	**99.45**
Lucerne	98.53	**99.78**
Wheat	97.23	**99.36**
Bare soil	99.01	**99.79**
Beet	91.93	**98.36**
Rape seed	97.02	**99.28**
Peas	93.79	**99.30**
Grass	94.32	**99.74**
OA	96.83	**99.45**
kappa	0.96	**0.99**

**Table 4:** Comparison of OAs of AIRSAR Flevoland (%).

	PauliRGB-CNN [26]	Dual-CNN [26]	RV-CNN [27]	CV-CNN [27]	FFS-CNN
OA	94.01	98.56	95.3	96.2	**99.45**

**Table 5:** The classification accuracies of GF-3 Wuhan (%).

Method	Water	Forest	Building	Farmland	OA	Kappa
Lenet-5 (Samples 1)	97.84	94.05	85.31	89.38	90.31	0.84
FFS-CNN (Samples 1)	**98.25**	**98.45**	**97.12**	**94.75**	**97.31**	**0.95**
Lenet-5 (Samples 2)	97.16	92.71	88.37	81.36	90.78	0.85
FFS-CNN (Samples 2)	97.74	97.70	95.70	87.51	95.63	0.92

**Table 6:** The classification OAs of FFS-CNN and decreased-feature-size CNN (%).

Dataset	FFS-CNN	Decreased-Feature-Size CNN
RS-2 Flevoland	94.40	92.78
AIRSAR Flevoland	99.45	94.34
GF-3 Wuhan	97.31	94.46

**Table 7:** The OAs of different training data (%).

Training Data Type	RS-2	RS-2	AIRSAR	AIRSAR	GF-3	GF-3
	Flevoland	Flevoland	Flevoland	Flevoland	Wuhan	Wuhan
	Lenet-5	FFS-CNN	Lenet-5	FFS-CNN	Lenet-5	FFS-CNN
Multi-class training samples	90.96	94.40	96.83	99.45	90.31	97.31
One-class training samples	89.58	91.23	95.53	97.12	91.94	95.93

**Table 8:** The relationship between overlap ratio and OAs (%).

Dataset	FFS-CNN					Lenet-5
	0	1/4	1/2	3/4	7/8	
RS-2 Flevoland	90.14	91.85	93.63	94.40	94.48	90.96
AIRSAR Flevoland	95.12	97.37	98.81	99.45	99.57	96.83
GF-3 Wuhan	94.65	95.80	96.90	97.31	97.38	90.31

**Table 9:** The relationship between overlap ratio and classification time (min).

Dataset	FFS-CNN					Lenet-5
	0	1/4	1/2	3/4	7/8	
RS-2 Flevoland	0.12	0.21	0.42	2.19	20.00	5.88
AIRSAR Flevoland	0.09	0.17	0.39	2.07	18.72	2.63
GF-3 Wuhan	0.08	0.12	0.28	1.39	12.35	2.88
